# Supplementary material for: Bayes-Like Integration of a New Sensory Skill with Vision
Source: Sci Rep. 2018 Nov 15;8:16880. doi: 10.1038/s41598-018-35046-7 (PMC6237778; doi:10.1038/s41598-018-35046-7)
Supplement: Supplementary file 2 — Supplementary Information [file 41598_2018_35046_MOESM2_ESM.pdf]

Bayes-Like Integration of a New Sensory Skill with Vision

James Negen[1*], Lisa Wen[1], Lore Thaler[1], and Marko Nardini[1]

[1]Department of Psychology, Durham University

Durham, Durham, UK, DH1 3LE

*Correspondence: jnegen@gmail.com or james.negen@durham.ac.uk

Supplemental Information

Additional Methods Details

This section gives additional details of the methods required for replication of the experiment.

Stimulus generation for sessions 3 through 5. Sessions 3 through 5 used a common scheme for generating trials that are audio-only, visual-only, or audio-visual. First we took the number of available trials, divided by three, and rounded down. We then generated this number of distinct audio-visual trials. For these, we selected centers for the visual stimuli that were even from 10 m to 35 m on a logarithmic scale. We calculated a bank of cumulative probabilities spaced evenly from 2% to 98%. Each visual center was paired randomly with a single cumulative probability. The actual target (Patchy's location) was then placed at the appropriate place on the visual cue's distribution according to its associated cumulative probability. For half of participants, on all of the trials in Session 3, and all but the last 10 trials of Session 4, the standard deviation on a log scale was 75% of the estimate of their audio standard deviation on a log scale as generated on the 290th trial of Session 2. For the rest of the trials, it was 125%. For the other half of participants, this was switched, with the higher standard deviation going first. Finally, an appropriately-delayed sound corresponding to the actual Patchy location was generated and paired to the target. These audio-visual trials were placed randomly in the available trial slots.

Training session 1. This session was designed to introduce the echo cue and scaffold participants' learning towards its use. As with all sessions, participants were greeted with Patchy saying "Let's echo! Press A!" which remained until the participant pressed A. He then said, "First just listen to the nearest and furthest places I can hide." Participants then listened and watched passively while Patchy moved back and forth between 10 m and 35 m distance five times, playing the correct sound with each appearance. He then said, "Try to find me! Use the left stick and A." Participants were then played an audio stimulus indicating either 10

m or 35 m. They then used the left joystick to select between those two options. If they were correct, Patchy would appear under their arrow and play an animation like nodding yes (up and down). If they were incorrect, Patchy would appear at the correct target and play an animation like shaking his head no (left and right). There were a total of 50 of these two-alternative forced-choice (2AFC) trials, with 25 of the targets at 35 m and 25 and 10 m.

When those 50 trials were completed, they moved on to a 3AFC version. The targets were at 10 m, 22.5 m, and 35 m. Patchy said, “Good job! Now listen to the middle too.” They were then played all three sounds, cycling from nearest to farthest and coinciding with Patchy’s appearance at those distances, four times. He then said, “Now try to find me!” and 100 trials of 3AFC were run. Feedback was given in the same way as the 2AFC. The distribution of targets was even among the three possible places.

When those 100 trials were completed, they moved on to a 5AFC version. The targets were at 10 m, 16.25 m, 22.5 m, 28.75 m, and 35 m. Patchy said, “Good job! Now listen to two more.” They were then played all five sounds in three cycles. He again said “Now try to find me!” and 150 trials of 5AFC were run. Feedback was the same again, and the target distribution was again even. No introduction of the next session was given. As with the ending of all sessions, Patchy said, “That’s all for today! Bye!” and the participant was helped out of the equipment.

Warmup block. Sessions 2 through 5 began with a warmup block that was a shortened version of Training Session 1. It was all the same except there were only 8 trials of 2AFC, 12 of 3AFC, and 20 of 5AFC, for a total of 40. This was done to remind participants of how the echo cues work.

Training session 2. This session was designed to help participants move from using the echoes for identifying a constrained set of possible responses over to making a response anywhere along a continuum. The session began with the warmup block for 40 trials and then

asked participants to find Patchy based on the audio cue alone, with feedback, for 250 trials. Target locations were spaced evenly from 10 m to 35 m on a logarithmic scale, shuffled into a random order. Every 50 trials, Patchy appeared briefly to tell participants their average error in percent over that period - this was in addition to being given the percent error after every trial. This was phrased as, “For echoes, you were off by X% on average over the last 50 trials.”

After completing the 290th trial of the session, two things happened. First, the standard deviation of participants’ errors (response minus target) over the last 100 trials was calculated after converting both targets and responses onto a log scale. A log conversion as used to account for the expected effects of Weber’s Law on time interval judgements[1]. This estimate of auditory-only performance was used to generate the trial parameters for the rest of the experiment as detailed below.

Second, the next session was previewed. The visual cue was explained to participants. Patchy said, “You’re almost done today. Let me show you what you’ll do next time. Let’s try something you can see. I like hiding by bubbles. More bubbles nearby means I’m more likely to be there. If you only see bubbles, the best you can do is point where there are the most of them.” No further instructions about the log-normal distribution were given. Participants were then given five opportunities to find Patchy with just the visual cue. They were given feedback on each trial. Then Patchy said, “From now on, sometimes you get both the echoes AND the bubbles!” Participants were then given five opportunities to find Patchy with both the audio and visual cues, again with feedback. Visual stimuli (bubble distributions) had a standard deviation on a log scale that was either 75% or 125% of the estimate of each participant’s audio-cue standard deviation (see above), with half of participants assigned to each of these conditions (see below). The centers of the bubble distributions were placed evenly on the response line on a log scale, in random order. The targets’ deviations were

generated as 10 values from -2 to +2 standard deviations and then randomly assigned to the distributions, with the actual target locations truncated at 10m and 35m. This completed session 2.

Main testing session (3). This session was intended to assess cue combination with the newly-learnt cue. It began with the warmup block for 40 trials and then had 249 trials that were a mix of audio-only, visual-only, and both. The trial parameters were generated as described above. Feedback was given after every trial and aggregate feedback was given every 50 trials, as described above. Patchy appeared briefly just before the first visual-only trial to say, “Remember those bubbles I like?”

For the last 10 trials, the next session was previewed. Patchy appeared and said, “Almost done today. Let’s look at what you’ll do next time. Let’s try an echo with a different sound going out. The way you use it is the same. More time between sounds means further out. For these, you won’t see me pop up.” They were then given 10 trials, evenly spaced on a log scale, with audio stimuli using the untrained frequency. When they entered a response, the arrow bobbed down into the sea for a quarter of a second, but they were not given any feedback (nor on any other trial involving the untrained frequency). This completed session 3.

Frequency generalization session (4). This session was designed to see if participants’ learning in the first session was specific to the emission that they learned or if it would generalize to a new frequency. It again started with the warmup block for 40 trials. Then there were 248 trials that went in the order of (i) a reinforced trial with the trained frequency, (ii) an unreinforced trial with just the audio at a new frequency, (iii) a reinforced trial with just a visual stimulus, and (iv) an unreinforced trial with the new frequency and the visual stimulus, repeating 62 times. The trials with the new frequency were generated in triplets as described above. This sequence was selected to keep the session from becoming

too discouraging and also to give participants some feedback to help keep them calibrated to the mapping of delays. Patchy appeared every 50 trials to give aggregate feedback as before, including the new frequency trials in the report.

For the last 10 trials, the next session was previewed. Patchy appeared and said, “The bubbles are going to be a little [more/less] spread out from now on.” Then there were 10 trials with the visual stimulus with a changed standard deviation, 75% or 125% of the estimate from above. The actual targets were evenly spaced from -2 standard deviations to 2 standard deviations, in random order, truncated at 10m and 35m. Feedback was given. This completed session 4.

Reliability generalization session (5). This session was designed to see if participants could adapt to a change in the reliability of one of the stimuli. First there was the warmup block of 40 trials. Then there were 10 trials, with feedback, with the visual stimulus and the changed reliability. This was done to make sure that participants could notice the change. A single audio-only trial was inserted to remind them that they needed to listen for it, with a target in the middle of the response line and feedback. Then there were 249 trials that were a mix of audio-only (with the trained frequency), visual-only with lower reliability, and both. Feedback was given on the visual-only trials and the audio-only trials, but not on trials with both cues. This was done to prevent more rote methods of adaptation. The trial parameters were generated in triplets as described above. Aggregate feedback was given every 50 trials for the audio-only trials. This completed the final session.

Table S1. Session organization for the Main Experiment.

Session	Stimulus	Response	No. of Trials	Feedback
1	Audio only	2AFC	50	Yes
1	Audio only	3AFC	100	Yes
1	Audio only	5AFC	150	Yes
2	Audio only	2AFC	8	Yes
2	Audio only	3AFC	12	Yes
2	Audio only	5AFC	20	Yes
2	Audio only	Continuous	250	Yes
2	Visual only	Continuous	5	Yes
2	Both	Continuous	5	Yes
3	Audio only	2AFC	8	Yes
3	Audio only	3AFC	12	Yes
3	Audio only	5AFC	20	Yes
3	Mixed: Audio, Visual, Both (83 each)	Continuous	249	Yes
3	Audio w/ new emission	Continuous	10	No
4	Audio only	2AFC	8	Yes
4	Audio only	3AFC	12	Yes
4	Audio only	5AFC	20	Yes
4	Mixed: Audio w/ trained emission, Audio w/ new emission, Visual, Both w/ new emission (62 each)	Continuous	248	Yes for audio w/ trained emission, Yes for visual, No for others
4	Visual w/ changed reliability	Continuous	10	Yes
5	Audio only	2AFC	8	Yes
5	Audio only	3AFC	12	Yes
5	Audio only	5AFC	20	Yes
5	Visual only w/ changed reliability	Continuous	10	Yes
5	Audio only	Continuous	1	Yes
5	Mixed: Audio, Visual w/ changed reliability, Both w/ changed visual reliability (83 each)	Continuous	249	Yes for audio only or visual only, No for both

Results of the Untrained Control Experiment

Comparing across the main and control experiments, the data suggest that the echo-like cue is genuinely a new augmented sensory skill and not an understanding of reflected sound that is acquired from everyday experience. In the training experiment, participants readily learned to estimate distances using the echo-like audio cue in the first two sessions. Each participant showed a significant correlation between target and response locations in the last half of the first session and also throughout the second session, all correlation coefficients $> .80$ (median $r = 0.87$), all p-values $< .001$ (individually displayed in S2). This is in line with previous findings that humans can quickly learn to use echoes and echo-like stimuli to make simple spatial judgements [2–4].

In contrast, in the control experiment, we did not find evidence that our echo-like cue was useful to untrained participants. To examine this formally, we reasoned that the cue could not be characterized as useful to the participants if they could not even outperform a simple strategy that did not depend at all on the stimulus: pointing to the center of the response line on every single trial. We regressed the squared distance between the target and the response (error) minus the squared distance from the target to the center (error predicted from just pointing to the center) onto the trial number (Figure S1). The fit line was above 0 for all trial numbers in the untrained group, and its 95% CI was above 0 until trial 189, never falling entirely below 0. That is, participants performed *worse* with the audio cue than if they had ignored the cue and simply pointed to the centre of the line. In comparison, the same line and 95% CI is below 0 for all trial numbers in the main experiment. This suggests that training allowed participants to outperform the degenerate strategy for this entire block of trials, but untrained people were worse for at least 189 trials. Mere exposure to the range of the stimuli may be responsible for a slow improvement that could potentially lead to some useful mapping of the cue over a longer time-scale, but crucially subjects with neither

feedback nor this exposure – the left-hand (trial 0) estimate for the regression line – do not show this.

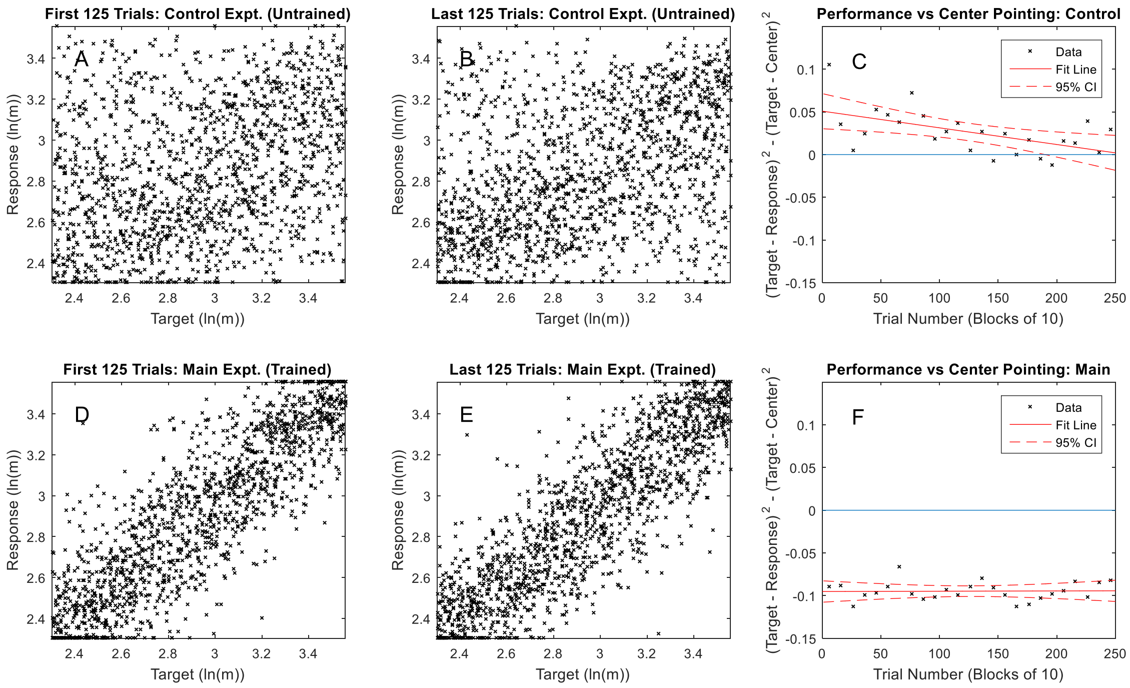

Figure S1. Comparing the Main and Control Experiments. The left four graphs show targets versus responses, broken down by the first 125 trials (A, D) versus the last 125 trials (B, E), and also untrained (A, B) versus trained (D, E). In the right column (C, F), the y-axis is the squared error on a log scale that the participant made minus the squared error that would be made by pointing just at the center of the response line regardless of the stimulus. The red slanted line is the fit to the data, the dashed lines are 95% confidence interval, the blue line is a reference at 0, and the black crosses are means for blocks of 10 trials. Data above the blue line indicate worse performance than the degenerate point-to-center strategy.

Supplemental Figures and Analysis for the Main Experiment

Variations on the Main Analysis. Several variations on the main four tests suggest that findings are consistent across several reasonable ways of analysing these data. The first three main tests all converge when total error (target minus response, squared) is entered instead of variable error: z = 2.48, p = .013 (5% to 26% TE Reduction) for Session 3; z = 3.00, p = .003 (10% to 32% TE Reduction) for Session 4; and z = 2.044, p = .041 (6% to 19% TE Reduction) for Session 5. They also converge when the variable error for each

participant/session/trial type is averaged over targets and entered by pairing different trial types with same participant and session (resulting in 12 pairs for each of the following): p = .032 (2% to 28% VE Reduction) for Session 3, p = .005 (6% to 31% VE Reduction) for Session 4, and p < .001 (6% to 17% VE Reduction) for Session 5. An analogous result happens for the fourth main test, averaging the visual reliance measures within each participant/session across targets, pairing the same participant's Session 3 and Session 5, p < .001 (Cohen's d = 1.65, 95% CI: 1.23 to 2.85). Under certain circumstances, we would also be able to use the constant error to further test for cue combination. However, given the similarity between constant errors in the two cues, t(35) = 1.70, p = .098 (95% CI: -.05 to .005), they cannot also be used this way; we would expect the constant error in the bimodal trials to be similar regardless of whether combination was occurring or not.

Full display of Echo-cue Performance by Participant and Session

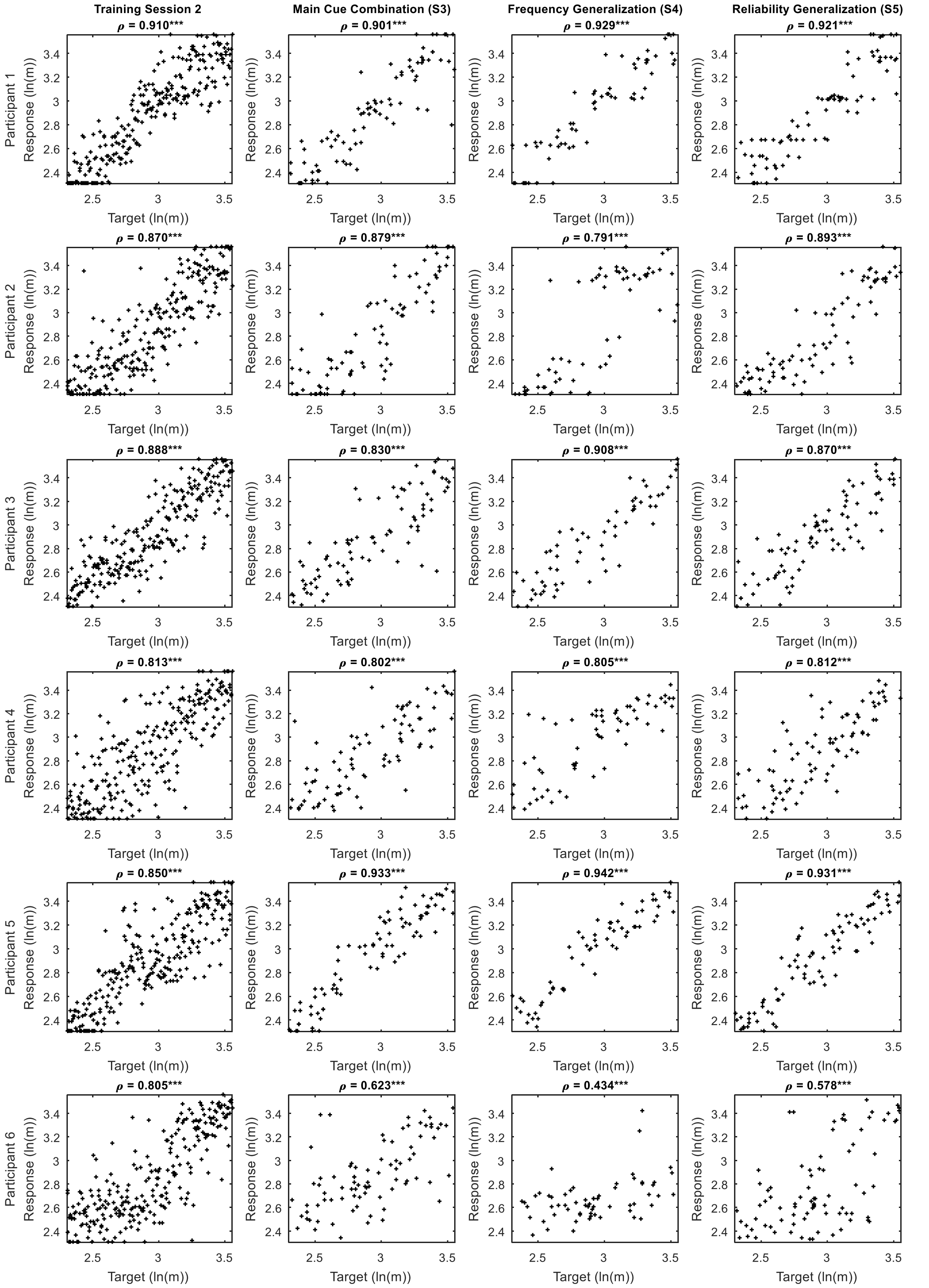

Figure S2. Targets versus responses for the audio-only trials in all sessions (2-5) with a continuous response (participants 1-6). For the frequency generalization sessions, the trials for the new frequency are displayed.

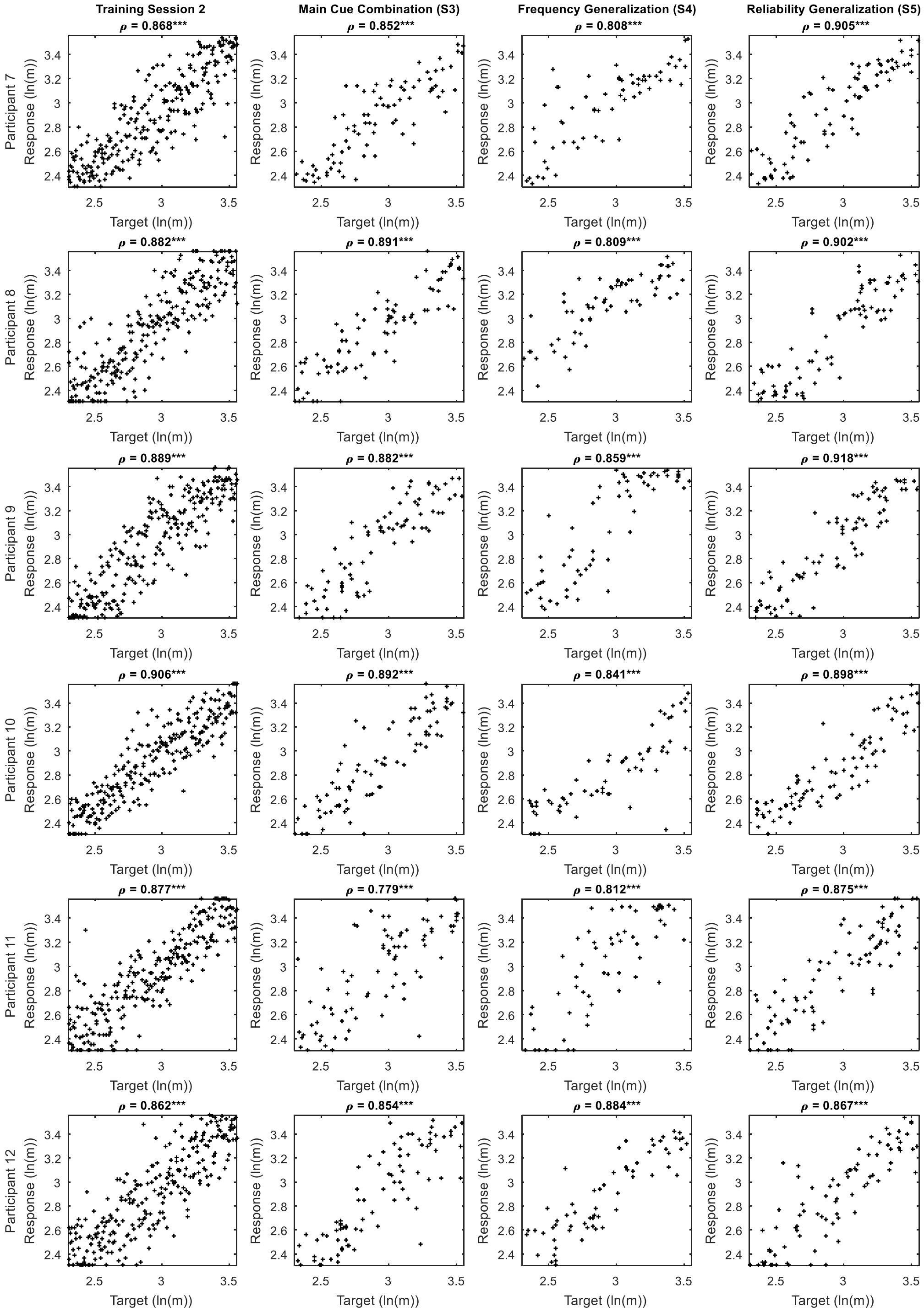

Figure S2 continued. Participants 7-12.

Full Display of Biases

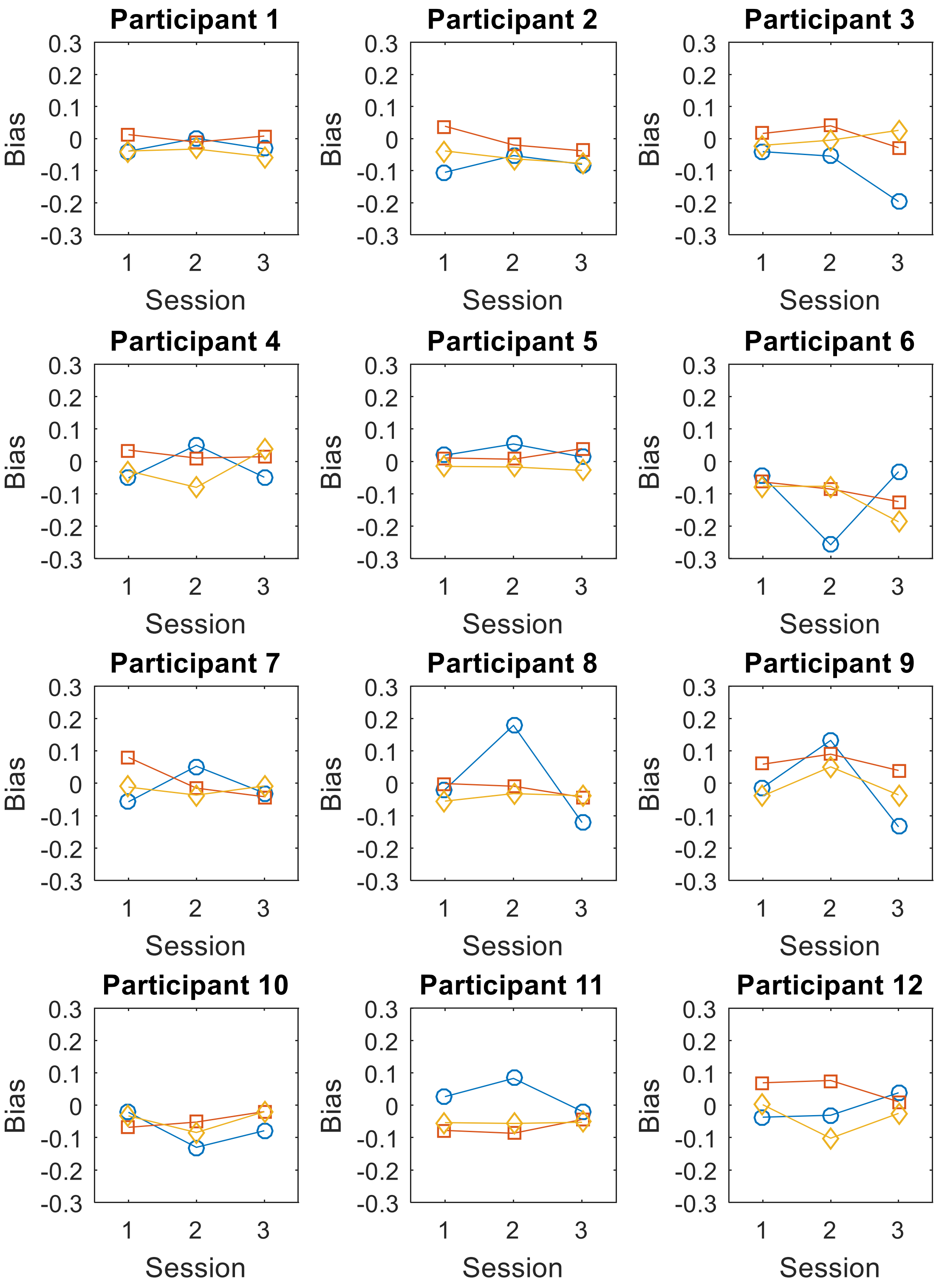

Figure S3. Bias for each participant (panels), session (x-axis), and trial type (audio in blue circles, visual in red squares, audio-visual in orange diamonds).

Second Experiment with the Vibrotactile Cue

Objective. To replicate the main experiment but with a different cue, one that was very different from echolocation but still a novel cue. This is important to see if the results in the main text are strictly unique to auditory or echo-like stimuli, or those linked to a natural physical relationship. Vibrotactile cues are an obvious choice since they are also used in successful sensory substitution methods[5].

Methods. Methods were as similar as possible in detail to the main experiment. The audio cue was replaced with a vibrotactile cue. This was made by harvesting the motor from a mobile phone, wiring it to an Arduino, and sewing it to a Velcro band (Figure S4). This could then be sent pulses of 0.0 to 5.0 volts by the computer running the VR simulation. The band was strapped around each participant’s left wrist. After piloting, we chose to use the range of 1.0 to 3.5 volts since perception of the intensity seemed roughly linear as a function of voltage. We however distorted the mapping logarithmically so that near distances were easier to discriminate than far distances, to be able to re-use the same analyses as the main text. This was done by spreading the voltages evenly on a logarithmic scale from ln(10m) to ln(35m). The pulses played for 1 second, embedded in 1.2 seconds of white noise delivered through headphones. There was no equivalent to the main text’s Session 4 (audio frequency generalization). There were 12 participants (3 male) with an age range of 19 to 24.

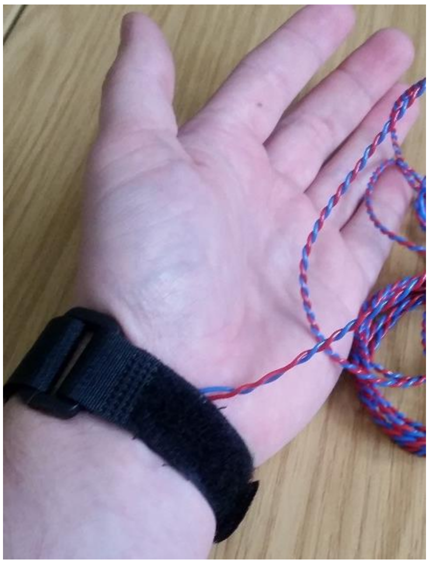

Figure S4. A photograph of the vibrotactile device as worn by a participant.

Results. Each of the most central analyses replicated here. The first criterion for Bayes-like cue combination was met by a reduction in variable error when comparing the best single cue to both cues in Session 3 (Figure 6 in main text), $z = 1.937$, $p = .026$, with a VE reduction of 9%, and in Session 4 when the visual reliability changed, $z = 3.289$, $p < .001$, with a VE reduction of 16%, both $N = 996$ pairs. The second criterion was met by a shift in reliance on vision as it became more/less reliable in the final session. Here the posterior mean weight change was estimated at 0.400, with a 95% credible interval of 0.058 to 0.732. As is the case with the main experiment, the raw data are also available in the attached supplements for further interrogation.

Discussion. While this is only one other kind of augmented cue, it does rule out the possibility that the main results are completely unique to echolocation. It also speaks to the reliability of the main findings.

Supplemental References

1. Getty, D. J. Discrimination of short temporal intervals: A comparison of two models. *Percept. Psychophys.* **18,** 1–8 (1975).
2. Teng, S. & Whitney, D. The acuity of echolocation: Spatial resolution in the sighted compared to expert performance. *J. Vis. Impair. Blind.* **105,** 20–32 (2011).
3. Tonelli, A., Brayda, L. & Gori, M. Investigate echolocation with non-disabled individuals. *J. Acoust. Soc. Am.* **141,** 3453–3453 (2017).
4. Thaler, L., Wilson, R. C. & Gee, B. K. Correlation between vividness of visual imagery and echolocation ability in sighted, echo-naïve people. *Exp. Brain Res.* **232,** 1915–1925 (2014).
5. Chebat, D.-R., Maidenbaum, S. & Amedi, A. Navigation Using Sensory Substitution in Real and Virtual Mazes. *PLoS One* **10,** e0126307 (2015).
